# Association Between Digital Device Utilization and Asthenopia Among Medical Students at King Khalid University

**DOI:** 10.7759/cureus.45621

**Published:** 2023-09-20

**Authors:** Abdulrahman Alamri, Manar M Alamri, Fatimah A Rashid, Amal S Alawashiz, Fatimah H Alqahtani, Alhanoof A Alyami, Lena A Almathami, Razan A Alwabel, Elham M Alqarni, Albatool M Alqahtani, Hanan Almoghamer, Abeer A Alahmari

**Affiliations:** 1 Ophthalmology, College of Medicine, King Khalid University, Abha, SAU; 2 Faculty of Medicine, King Khalid University, Abha, SAU

**Keywords:** medical students, ocular problems, saudi arabia, digital devices, asthenopia

## Abstract

Background

Digital eye strain has become a serious concern due to the exponential increase in the usage of digital devices in recent years. This study aimed to assess the prevalence of digital eye strain among medical students.

Methods

A cross-sectional study was conducted over a period of one month, from 15th June to 15th July 2023, among undergraduate medical students at King Khalid University, Aseer Region, Saudi Arabia. An online survey was carried out using a structured questionnaire that was circulated through social media platforms (Facebook, Messenger, Instagram, Telegram, and WhatsApp).

Results

A total of 300 students were enrolled in this study. The prevalence of ocular problems, in descending order, was as follows: asthenopia had the highest prevalence at 30.5% (94), followed by conjunctivitis at 9.4% (29); squint, glaucoma, and cataract had lower prevalence rates of 3.9% (12), 1.6% (5), and 1.6% (5), respectively. The majority of participants used digital devices for fun (92.9% (286)) and study (95.5% (294)). Significant positive correlations were found between asthenopia and the hours spent on digital devices for studying (r = 0.161, p = 0.005), communication (r = 0.146, p = 0.011), and entertainment (r = 0.206, p < 0.001).

Conclusions

A substantial number of medical students are experiencing asthenopia. Prolonged usage of these devices is linked to a higher prevalence of asthenopia.

## Introduction

The evaluation of technology in education has brought about a noticeable transformation in the methods of teaching, presenting information, and sources for studying. Students prefer using laptops, tablets, and cell phones, not just for recreational purposes but also for their academic activities, to avoid the burden of carrying heavy books [1]. Moreover, the coronavirus disease 2019 (COVID-19) pandemic has significantly impacted the traditional education system. Students reported several preferred aspects of online education, including teachers' support with electronic resources, the use of online platforms for accessing resources and tests, easier and individualized communication with teachers, and peer connectivity for collaborative projects [2]. However, this convenient lifestyle has also raised health-related concerns [3].

Digital eye strain has become a serious concern due to the exponential increase in the usage of digital devices in recent years. Several individuals suffer from physical discomfort, such as vague pain in the eyes, neck, head, and shoulders, as well as dryness, blurring, and watering of the eyes after screen use for longer than two hours at a time. This collection of symptoms is referred to as digital eye strain. The American Optometric Association defines computer vision syndrome as "a complex of eye and vision problems related to near work experienced during computer use" [4]. The pathophysiology of digital eye strain involves multiple factors contributing to its development, including reduced contrast levels of letters on digital screens compared to the background, screen glare and reflections, incorrect distance and angle of viewing screens, inadequate lighting conditions, improper posture during usage, and infrequent blinking of eyes [5]. It is estimated that globally around 60 million people suffer from this syndrome [6]. According to the Digital Eye Strain Report of 2016, which surveyed over 10,000 adults from the United States (US), the overall self-reported prevalence of digital eye strain was 65%. The report also revealed that females were more commonly affected than males, with a prevalence of 69% compared to 60% [7].

Undergraduate medical students, hailing from different parts of the world and staying at hostels away from their homes at a tender age, inevitably embrace digital devices to communicate with family, socialize with peers, play games to beat stress, engage in academic activities like preparing for seminars and symposiums, and keeping themselves updated with recent advances in the medical field [3]. The rationale for this study is based on the limited existing research on digital eye strain specifically among medical students in Saudi Arabia. As far as we know, there are no studies conducted in the Aseer Region to assess the prevalence of digital eye strain among medical students.

## Materials and methods

A cross-sectional study was conducted over a period of one month, from 15th June to 15th July 2023, among undergraduate medical students at King Khalid University, Aseer Region, Saudi Arabia. An online survey was carried out using a structured questionnaire that was circulated through social media platforms (Facebook, Messenger, Instagram, Telegram, and WhatsApp) to undergraduate medical students. The questionnaire was distributed online by loading it onto Google Forms. Participants were able to access and respond to the questionnaire through the Google Forms platform.

Sample size calculations for this study were performed using G*Power (https://www.psychologie.hhu.de/arbeitsgruppen/allgemeine-psychologie-und-arbeitspsychologie/gpower). With a two-tailed test, an effect size of 0.1, a significance level of 0.05, and a desired power of 0.90, a minimum of 263 participants per group was determined to be necessary. To account for potential non-response and incomplete or inconsistent data, the sample size was increased to 300 participants, with a mean age of 19.3± 3.7. Females represented 47.0%. The study population was selected to ensure that it represents the target population to control bias. The majority (98.7%) did not suffer from chronic diseases. The study participants were collected through non-random sampling methods, specifically using convenience sampling and snowball sampling.

The study included health students aged 18 years or above who were studying at King Khalid University. However, to ensure a focused and relevant sample, we excluded individuals with any communication disability, such as being deaf or mute, as well as students with specific eye conditions like corneal dystrophies and degeneration, keratoconus, family history of ocular disease, hereditary diseases of the eye, conjunctivitis, diabetes, and hypertension.

The questionnaire was created by the authors, and it consists of two sections: section one consisted of nine questions designed to gather comprehensive information from the study participants, who were medical students at King Khalid University. The first question verifies the participants' status as medical students at the university. The second question inquiries about their preferred study method, with options for studying from printed papers and books, using tablets and laptops, or specifying other methods if applicable. The following questions focus on the participants' eye health: question three assesses the presence of asthenopia (asthenopia encompasses various symptoms related to eye usage, commonly including eyestrain, headaches, and feelings of tiredness or soreness in the eyes or eyelids), question four asks about recent eye inflammations (conjunctivitis) or infections, and question five explores preexisting medical conditions such as arthritis, osteoporosis, thyroid disease, diabetes, hypertension, chronic migraine, or chronic headache. Moving on, question six queries whether participants have strabismus, and question seven investigates if they have glaucoma. Question eight inquiries about the presence of cataracts. Lastly, question nine asks participants if they have undergone any eye surgery, providing an option for them to specify the type of surgery if applicable.

Section 2 consisted of six questions focused on the participants' digital device usage and reasons for using them. Question 1 asks about the number of hours spent on digital devices for studying, with options ranging from less than 2 hours to over 6 hours. Question 2 assesses the number of hours spent on digital devices for communication, again with multiple time-based options. Question 3 inquiries about the hours spent on digital devices for entertainment, offering several time-based choices. Question 4 specifically addresses the hours spent using screens in a dark room, with similar time-based options. Question 5 asks participants if they wear glasses, contact lenses, or none of these options. Lastly, question 6 requires participants to choose all the reasons they use digital devices, which include studying, work, entertainment, and communication.

The study ensured that written consent was obtained from all participants before they participated in the research. Ethical clearance was obtained from both King Khalid University and the Kingdom of Saudi Arabia's Ministry of Education (IRB HAPO-06-B-001). The study adhered to the guidelines of the Helsinki Declaration to uphold ethical principles in medical research involving human subjects. Data collection was conducted anonymously, and participants' confidentiality was rigorously maintained throughout the study.

The collected questionnaire data were analyzed using two statistical software: R version 4.2.1 (R Foundation for Statistical Computing, Vienna, Austria) and Microsoft Excel (Microsoft Corporation, Redmond, WA). For categorical variables, the analysis involved representing the data in terms of frequency and percentages. To investigate the dependency between asthenopia and digital device use, we used a negative biserial correlation analysis with a p-value of less than 0.05 indicating statistical significance.

## Results

**Figure 1:**
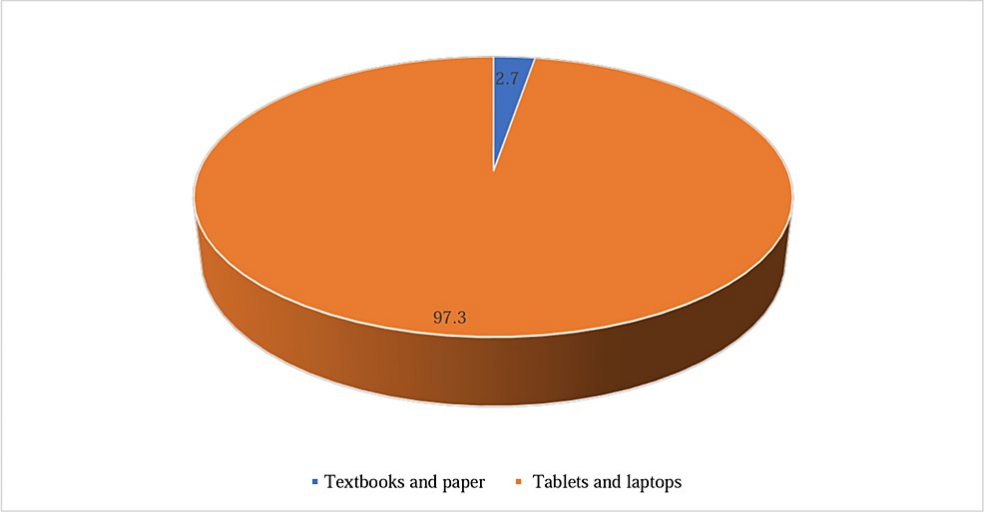
Preferred study tools: tablets and laptops vs. textbooks and paper

Figure 1 provides information on the preferred devices used by participants for studying. It shows that the majority of participants (97.3%; 292/300) preferred using modern devices like tablets and laptops. On the other hand, a smaller proportion of participants (2.7%; 8/300) preferred using traditional methods such as textbooks and paper for their studies.

**Figure 2:**
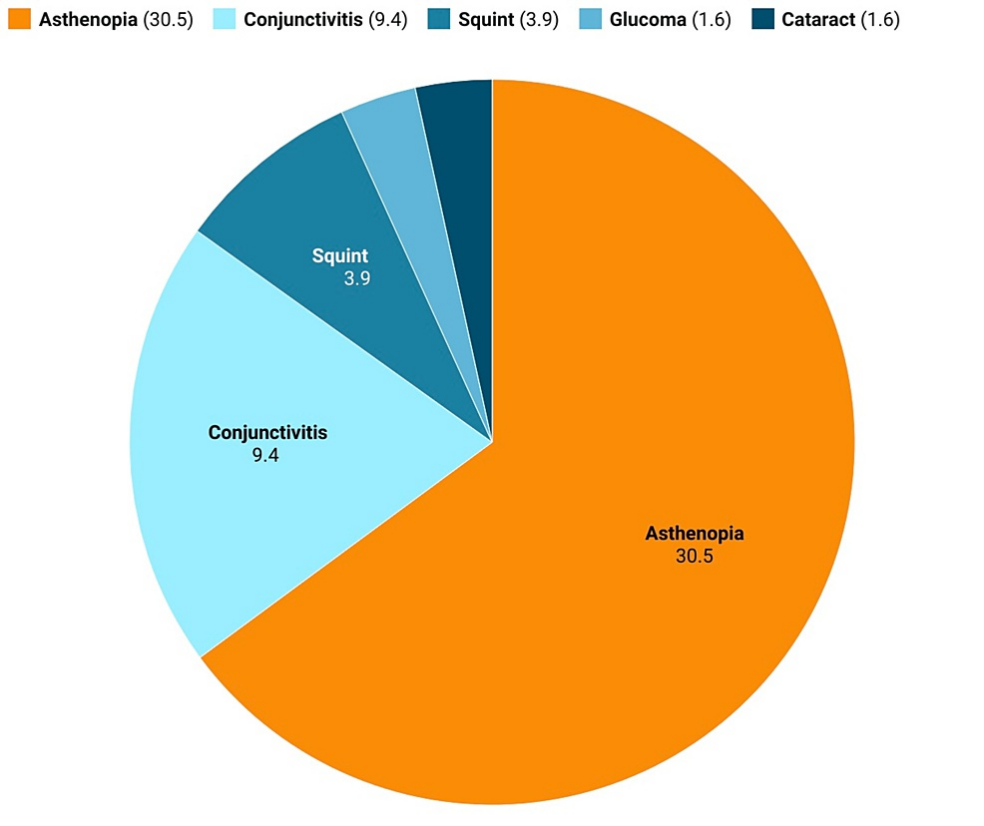
Prevalence of eye conditions among study participants

Figure 2 presents the prevalence of different eye conditions among the study participants. Asthenopia has the highest prevalence at 30.5%, followed by conjunctivitis at 9.4%. Squint, glaucoma, and cataract have lower prevalence rates of 3.9%, 1.6%, and 1.6%, respectively.

Table 1 provides valuable insights into the participants' digital device usage patterns for studying, communication, entertainment, and screen usage in a dark room. For studying, nearly two-thirds of participants (65.3%) spend more than two hours on digital devices, with 35.1% spending over six hours. Regarding communication, a significant number of participants (55.2%) use digital devices for less than two hours while only 6.2% use them for more than six hours. Regarding entertainment, the majority (61.8%) spend between two and six hours on digital devices, with 40.9% spending two to four hours. For screen usage in a dark room, over half of the participants (51.9%) spend less than two hours.

**Table 1:** Digital device usage patterns and screen time habits among participants

Number of hours		Frequency	Percent
Spent on digital devices for studying	Less than 2 hours	9	2.9
	Between 2 and 4 hours	89	28.9
	Between 4 and 6 hours	93	30.2
	More than 6 hours	108	35.1
Spent on digital devices for communication	Less than 2 hours	170	55.2
	Between 2 and 4 hours	65	21.1
	Between 4 and 6 hours	45	14.6
	More than 6 hours	19	6.2
Spent on digital devices for entertainment	Less than 2 hours	52	16.9
	Between 2 and 4 hours	126	40.9
	Between 4 and 6 hours	72	23.4
	More than 6 hours	49	15.9
Spent using the screen in a dark room	Less than 2 hours	160	51.9
	Between 2 and 4 hours	84	27.3
	Between 4 and 6 hours	37	12.0
	More than 6 hours	18	5.8

**Figure 3:**
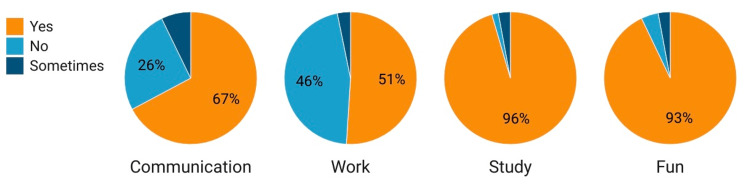
Reasons for using digital devices among medical students

Figure 3 presents data on participants' responses to different activities based on whether they find them fun, study-related, work-related, or communication-related. For the activity "Fun," the majority of participants (92.9%) responded "Yes"; only a small proportion (4.2%) responded "No." Regarding "Study," the overwhelming majority (95.5%) of participants responded "Yes"; a minimal percentage (1.6%) responded "No." For "Work," roughly half of the participants (51.0%) responded "Yes,". Meanwhile, 45.8% responded "No." Regarding "Communication," a significant majority (67.2%) of participants responded "Yes," indicating that they are involved in communication-related activities. A smaller proportion (25.6%) responded "No."

Table 2 presents data on participants' eye-related characteristics, specifically regarding their use of glasses or contact lenses and any history of ocular surgery. For wearing corrective eyewear, the majority of participants (53.6%) reported wearing glasses while a smaller proportion (10.1%) used contact lenses. About one-third of the participants (33.4%) indicated that they did not wear glasses or contact lenses. Regarding ocular surgery, the vast majority of participants (92.9%) responded "No." A small percentage (4.5%) reported having had ocular surgery.

**Table 2:** Ocular characteristics and eyewear usage among participants

		Frequency	Percent
Eyewear	Don’t wear glasses or lens	103	33.4
	Wear glasses	165	53.6
	Wear Contact lens	31	10.1
Ocular surgery	No	286	92.9
	Yes	14	4.5

Significant positive correlations were found between asthenopia and the hours spent on digital devices for studying (r = 0.161, p = 0.005), communication (r = 0.146, p = 0.011), and entertainment (r = 0.206, p < 0.001) (Table 3).

**Table 3:** Correlation analysis of asthenopia and digital device usage among university students r = correlation, p = p-value, * = significant (less than 0.05), ** = highly significant (less than 0.01)

		Asthenopia	Hours spent on digital devices for studying	Hours spent on digital devices for communication	Hours spent on digital devices for entertainment	Hours spent using the screen in a dark room
	r	1	.161**	.146*	.206**	0.081
	p	-	0.005	0.011	0	0.165
Hours spent on digital devices for studying	r	-	1	0.069	.129*	.144*
	p	-	-	0.231	0.026	0.013
Hours spent on digital devices for communication	r	-	-	1	.205**	.345**
Hours spent on digital devices for entertainment	r	-	-	-	1	.381**
	r	-	-	-	-	299
Hours spent using the screen in a dark room	p	-	-	-	-	1

## Discussion

As far as we are aware, this study is pioneering research to investigate the prevalence of asthenopia and examine its associated risk factors among medical students in the Asser region of Saudi Arabia. Furthermore, this study correlated digital device use with asthenopia. The majority of participants reported using digital devices for studying while nearly one-third had complaints related to asthenopia. Most participants utilized these devices for both fun and studying. The duration of device usage varied depending on the purpose. Approximately one-third of participants used digital devices for entertainment for more than six hours, whereas around half of them used them for communication for less than three hours. The findings suggest that many participants did not find value and comfort in using physical textbooks and paper for their studies. These insights could be beneficial for educators, institutions, and educational technology developers in tailoring their approaches to accommodate digital learning preferences and optimizing the learning experience for students.

In this study, we found that around one-third of the included medical students were complaining of asthenopia. Sawaya et al. conducted a cross-sectional study conducted at the American University of Beirut aimed to determine the prevalence of asthenopia (eye strain) among university students from various majors and identify its risk factors [8]. The study found a prevalence of 67.8% for asthenopia, with blurred vision being the most common symptom. A similar finding was reported in Iran. Hashemi et al. reported a prevalence of 49.4% for asthenopia [9]. Additionally, high rates of asthenopia were found among college students in several countries [5], including China with 53.5% [10], Malaysia with 89.9% [11], Egypt with 86% [12], Saudi Arabia with 74.96% [13], and a more recent study in Iran (70.9%) [14]. Asthenopia can arise from activities that demand prolonged visual focus such as reading or extended use of digital devices. We found that most of the participants who used digital devices for a prolonged duration had asthenopia. Likewise, during the COVID-19 pandemic, studies conducted among school children revealed a higher incidence of asthenopia when they spent longer hours on screen and engaged in online courses [15,16]. On the other hand, many studies reported that there was no statistically significant association between asthenopia and the amount of time spent using digital devices [13,17]. There was no statistically significant association between asthenopia and the amount of time spent using digital devices.

In this study, we did not find a significant relationship between asthenopia and digital device use in the dark. A similar finding was reported by Sawaya et al. [8]. This finding is noteworthy, especially considering recent research by the optical chemistry team at the University of Toledo, which described the detrimental effect of blue light emitted from digital displays [18]. It is essential to consider various factors, such as the wavelength, intensity, and duration of blue light exposure while assessing the impact of this type of light on eye health [19]. Additional investigations are warranted to gain a comprehensive understanding of how blue light exposure may influence asthenopia and other visual discomforts experienced by individuals using digital devices in the dark.

Strengths and limitations

A key strength is the comprehensive questionnaire covers various aspects of device usage, providing valuable information on usage duration and purposes. However, the study's limitations include a relatively small sample size, potentially limiting the findings' applicability to a larger population. Self-reported data could introduce biases in responses, and the cross-sectional design restricts the ability to establish causal relationships and observe changes in device usage patterns over time. We used a non-random sampling method, that may hinder the generalization of the findings. Finally, we used a questionnaire created by us, which has not been validated to collect our data. Despite these limitations, the study's insightful findings offer practical implications for educators and healthcare professionals, aiding in understanding and managing digital device usage patterns among medical students and informing potential interventions to promote eye health and responsible device use.

## Conclusions

This study provides valuable insights into the diverse utilization of digital devices among medical students, underscoring the importance of comprehending and effectively managing device usage patterns for different purposes. A substantial number of medical students are experiencing asthenopia. Prolonged usage of these devices is linked to a higher prevalence of asthenopia. In future research, incorporating qualitative approaches could enhance the interpretation of the results, delving into the reasons behind their preferences and exploring factors influencing their choices. Such qualitative insights would complement the existing findings and aid in formulating targeted interventions to promote responsible and eye-healthy digital device usage among medical students.
